# Multicenter Study of Azole-Resistant *Aspergillus fumigatus* Clinical Isolates, Taiwan^1^

**DOI:** 10.3201/eid2604.190840

**Published:** 2020-04

**Authors:** Chi-Jung Wu, Wei-Lun Liu, Chih-Cheng Lai, Chien-Ming Chao, Wen-Chien Ko, Hsuan-Chen Wang, Ching-Tzu Dai, Ming-I Hsieh, Pui-Ching Choi, Jia-Ling Yang, Yee-Chun Chen

**Affiliations:** National Health Research Institutes, Zhunan, Taiwan (C.-J. Wu, H.-C. Wang, M.-I. Hsieh, Y.-C. Chen);; National Cheng Kung University Hospital and College of Medicine, Tainan, Taiwan (C.-J. Wu, W.-C. Ko);; Fu Jen Catholic University Hospital and College of Medicine, New Taipei, Taiwan (W.-L. Liu), Chi Mei Medical Center, Tainan (C.-C. Lai, C.-M. Chao);; National Taiwan University Hospital and College of Medicine, Taipei, Taiwan (C.-T. Dai, P.-C. Choi, J.-L. Yang, Y.-C. Chen)

**Keywords:** Aspergillus fumigatus, fungi, antimicrobial resistance, azole resistance, azole use, clinical isolates, cdr1B, cyp51A, HMG-CoA reductase, TR_34_/L98H, TR_46_/Y121F/T289A, environments, humans, Taiwan

## Abstract

In a multicenter study, we determined a prevalence rate of 4% for azole-resistant *Aspergillus fumigatus* in Taiwan. Resistance emerged mainly from the environment (TR_34_/L98H, TR_34_/L98H/S297T/F495I, and TR_46_/Y121F/T289A mutations) but occasionally during azole treatment. A high mortality rate observed for azole-resistant aspergillosis necessitates diagnostic stewardship in healthcare and antifungal stewardship in the environment.

Worldwide emergence of azole-resistant *Aspergillus fumigatus* since the late 2000s threatens human health (*1*). Azole resistance in *A. fumigatus* might develop during patient therapy with medical azoles or through exposure to azole fungicides in the environment; environmental exposure predominantly involves TR_34_/L98H and TR_46_/Y121F/T289A mutations in *cyp51A* (*1*).

Taiwan is an island country in eastern Asia that is geographically separated from mainland Eurasia and has a long history of azole fungicide use. To delineate the influence of clinical and environmental use of azoles on resistance, we conducted a multicenter study that investigated 375 *A. fumigatus* sensu stricto isolates collected during August 2011–March 2018 from 297 patients at 11 hospitals in Taiwan (Appendix Table 1, Figure 1).

We confirmed the presence of azole resistance by using the Clinical Laboratory Standard Institute method (Appendix Table 1) (*2*). Isolates resistant to >1 medical azoles (itraconazole, voriconazole, posaconazole, and isavuconazole) were defined as azole-resistant *A. fumigatus* and examined for resistance mechanisms, microsatellite-based phylogenetic relatedness, and growth rates following previously described methods (*3*,*4*).

Overall, 19 isolates from 12 patients were azole-resistant *A. fumigatus*. These isolates had resistance rates of 4.0%/patient and 5.1%/isolate analyses (Appendix Tables 2, 3). Ten (83.3%) patients harbored azole-resistant *A. fumigatus* that had environmental mutations, including TR_34_/L98H (5 isolates, 5 patients), TR_34_/L98H/S297T/F495I (7 isolates, 4 patients), and TR_46_/Y121F/T289A (1 isolate) mutations. This observation is consistent with the estimated global prevalence of azole resistance in *Aspergillus* (3%–6%) and the predominance of environmental resistance mechanisms in azole-resistant *A. fumigatus* (*1*,*5*).

Phylogenetic analysis showed that TR_34_/L98H/S297T/F495I isolates from 2 patients with pulmonary aspergillosis (isolates B44 and B51 in 2012, isolates E071, E073, and E074 in 2015) (Figure) belonged to a local microsatellite genotype widely distributed in the environment of Taiwan (*3*), indicating that this clone has locally evolved and adapted to the environment. The TR_34_/L98H isolates were genetically clustered with local environmental isolates or clinical isolates from China and Europe (Appendix Table 4). The TR_46_/Y121F/T289A isolate (S05–322) recovered in 2018, which colonized a patient without overseas travel, was genetically identical to a clone prevalent in the Netherlands and Tanzania (*6*), raising the concern of the intercountry transfer of resistant isolates.

**Figure:**
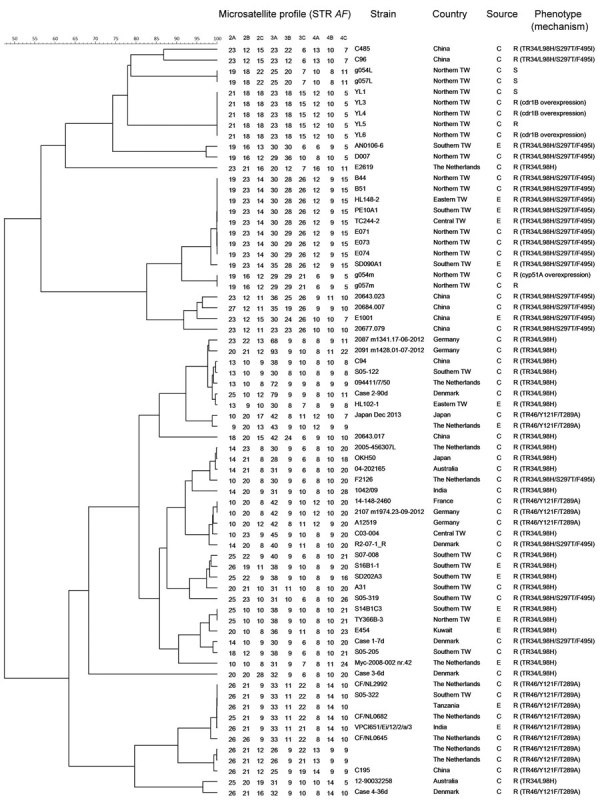
Genetic relatedness among *Aspergillus fumigatus* isolates based on microsatellite genotyping, Taiwan. Scale bar indicates percentage relatedness. *AF*, *A. fumigatus*; C, clinical; E, environmental; R, azole-resistant; S, azole-susceptible; STR, short tandem repeat; TW, Taiwan.

All TR_34_/L98H/S297T/F495I, TR_34_/L98H, and TR_46_/Y121F/T289A isolates exhibited cross-resistance to difenoconazole and tebuconazole (both triazole fungicides) without fitness cost, demonstrated by normal growth rates (Appendix Figure 2). The TR_34_/L98H/S297T/F495I isolates and TR_46_/Y121F/T289A isolates were also resistant to prochloraz (an imidazole fungicide) (Appendix Table 2). The prevalence of TR_34_/L98H/S297T/F495I isolates in Taiwan might be attributed to widespread use of prochloraz over the past 3 decades. Studies have suggested an association between use of imidazole fungicides and emergence of azole-resistant *A. fumigatus* with TR_34_/L98H/S297T/F495I mutations (*7*,*8*).

In Taiwan, the annual consumption of difenoconazole and tebuconazole has exceeded that of prochloraz since 2012 (Appendix Figure 3), further creating a favorable environment for maintenance and spread of TR_34_/L98H, TR_34_/L98H/S297T/F495I, and TR_46_/Y121F/T289A isolates. Thus, the One Health approach to implement environmental antifungal stewardship is warranted to minimize ongoing resistance selection in the fields.

Six azole-resistant *A. fumigatus* isolates with wild-type *cyp51A* were obtained from 2 patients. Four pan–azole-resistant urinary isolates were sequentially recovered from a patient (no. 11) with *A. fumigatus* renal abscesses who was receiving voriconazole for >3 months in whom an initial urine isolate was susceptible to azole; all 5 isolates were genetically identical.

Overexpression of *cdr1B* (a drug efflux transporter) and an S269P mutation in *hmg1* (a hydroxymethylglutaryl-CoA reductase) were identified in 4 resistant isolates but not in the initial susceptible isolate (Appendix Table 5, Figure 4), suggesting their roles involved in azole resistance (*4*,*9*). Another 2 pan–azole-resistant respiratory isolates were recovered from a patient (no. 12) who had pulmonary aspergillosis and was receiving voriconazole for 4 months. Azole-susceptible and azole-resistant isolates co-existed in this patient, which echoes the international recommendation suggesting testing multiple colonies (>5) from a single culture (*1*). *Cyp51A* overexpression and an F262 deletion in *hmg1*(*hmg1*^F262_del^) were identified in these 2 resistant isolates. Although *hmg1*^F261_del^ was recently reported in azole-resistant *A. fumigatus* from a voriconazole-exposed patient (*4*), whether *cyp51A* overexpression and *hmg1*^F262_del^ act synergistically to cause resistance warrants further studies.

Finally, reduced colony sizes were observed in all 6 azole-resistant *A. fumigatus* isolates with wild-type *cyp51A* (Appendix Figure 2). Thus, attention should be paid to select colonies of various sizes for susceptibility testing from patients with azole exposure.

Overall, 4 patients harboring azole-resistant *A. fumigatus* with environmental mutations and 2 patients harboring azole-resistant *A. fumigatus* with wild-type *cyp51A* showed development of invasive aspergillosis, and all had aspergillosis-related deaths. High mortality rates for azole-resistant aspergillosis we observed (6/6, 100%) and for those from a previous report (*10*) emphasize the need for a proposed integrated algorithm for management and control of azole-resistant aspergillosis (Appendix Table 6).

In conclusion, we report a health threat that arose from clinical and environmental use of azoles; environmental use contributed at a larger and global scale. These data necessitate diagnostic stewardship in the clinic and antifungal stewardship in the environment.

Appendix Additional information on multicenter study of azole-resistant *Aspergillus fumigatus* clinical isolates, Taiwan.
