# Supplementary material for: Multicenter Study of Azole-Resistant Aspergillus fumigatus Clinical Isolates, Taiwan
Source: Emerg Infect Dis. 2020 Apr;26(4):806–9. doi: 10.3201/eid2604.190840 (PMC7101115; doi:10.3201/eid2604.190840)
Supplement: Appendix — Additional information on multicenter study of azole-resistant Aspergillus fumigatus clinical isolates, Taiwan. [file 19-0840-Techapp-s1.pdf]

Article DOI: https://doi.org/10.3201/eid2604.19.0840

Multicenter Study of Azole-Resistant *Aspergillus fumigatus* Clinical Isolates, Taiwan

Appendix

Appendix Table 1. Details of participating hospitals, antifungal susceptibility testing, and isolate collection for analysis of azole-resistant *Aspergillus fumigatus* clinical isolates, Taiwan*

			Susceptibility testing method†	
Hospital	Location	Period of collection	Isolates before June 2017	Isolates during June 2017–March 2018
Chi-Mei Medical Center, Luiying (CMMC)	Southern	2015 Feb–2018 Mar	CLSI M38-A2	Screening azole agar plates; confirmed by CLSI M38-A2
National Cheng-Kung University Hospital (NCKUH)	Southern	2011 Aug–2018 Mar	CLSI M38-A2	Screening azole agar plates; confirmed by CLSI M38-A2
National Taiwan University Hospital (NTUH)	Northern	2012 Feb–2018 Mar	YeastOne	Screening azole agar plates; confirmed by CLSI M38-A2
TSARM hospitals				
Changhua Christian Hospital	Central	2016 Jul–Sep	CLSI M38-A2	NI
Ditmanson Medical Foundation Chia-Yi Christian Hospital	Southern	2016 Jul–Sep	CLSI M38-A2	NI
Hualien Tzu Chi Hospital	Eastern	2016 Jul–Sep	CLSI M38-A2	NI
Kaohsiung Medical University Chung-Ho Memorial Hospital	Southern	2016 Jul–Sep	CLSI M38-A2	NI
Kaohsiung Veterans General Hospital	Southern	2016 Jul–Sep	CLSI M38-A2	NI
Show Chwan Memorial Hospital	Central	2016 Jul–Sep	CLSI M38-A2	NI
Taichung Veterans General Hospital	Central	2016 Jul–Sep	CLSI M38-A2	NI
Tainan Sin Lau Hospital	Southern	2016 Jul–Sep	CLSI M38-A2	NI

*This study was approved by the Institutional Review Boards of the National Health Research Institutes (no. EC1040502-E and EC1050307) and participating hospitals: CMMC (10607-L01 and 10711-L03), NCKUH (B-ER-101–342), and NTUH (201605098RIPA). NI, no isolate; TSARM, Taiwan Surveillance of Antimicrobial Resistance of Molds.

†*A. fumigatus* sensu stricto was identified on the basis of morphologic characteristics, growth at 50°C, and sequence analyses of the internal transcribed spacer region and calmodulin gene (*1*). For isolates from CMMC, NCKUH, and TSARM hospitals, MICs of antifungal agents were determined by using the Clinical and Laboratory Standards Institute (CLSI) M38-A2 method. The MICs for the isolates from NTUH were determined by using the Sensititer YeastOne method (Trek Diagnostic Systems, http://www.trekds.com); isolates with any of the following MIC values (i.e., ≥2, ≥2, and ≥0.25 µg/mL of itraconazole, voriconazole, and posaconazole, respectively) were reexamined by using the CLSI method. For isolates collected during June 2017–March 2018, azole resistance was screened by using azole-containing agar plates. In brief, the conidia of these isolates were directly inoculated onto 3 Sabouraud dextrose agar plates supplemented with itraconazole (2 µg/mL), voriconazole (1 µg/mL), or posaconazole (0.25 µg/mL), and incubated at 37°C. Colonies that grew after 2–4 d on any of the azole-containing agar plates were selected for the MIC determination by using the CLSI method.

Appendix Table 2. Laboratory characteristics of *Aspergillus fumigatus* clinical isolates from 12 patients with aspergillosis, Taiwan*

Patient no.	Strain no.	Year (day)†	*Cyp51A* mutation	MIC or MIC_{50}/MIC_{90} (range), µg/mL, by CLSI M38-A2‡ AMB	ITR	VRC	POS	ISA	DFC	TBC	PRC
Azole-resistant isolates§ except YL1, g054L, and g057L											
1	B44	2012 (d0)	TR_{34}/L98H/S297T/F495I	0.5	>16	2	1	>16	>32	16	>32
1	B51	2012 (d2)	TR_{34}/L98H/S297T/F495I	0.5	>16	2	1	>16	>32	16	>32
2	D007	2014	TR_{34}/L98H/S297T/F495I	0.5	>16	2	1	>16	>32	>32	>32
3	E071	2015 (d0)	TR_{34}/L98H/S297T/F495I	0.5	>16	2	1	>16	>32	>32	>32
3	E073	2015 (d3)	TR_{34}/L98H/S297T/F495I	0.5	>16	2	1	>16	>32	>32	>32
3	E074	2015 (d4)	TR_{34}/L98H/S297T/F495I	0.5	>16	2	1	>16	>32	>32	>32
4	S05–319	2018	TR_{34}/L98H/S297T/F495I	0.5	>16	2	1	>16	>32	>32	>32
5	A31	2013	TR_{34}/L98H	0.5	>16	4	1	16	>32	32	4
6	S05–122	2016	TR_{34}/L98H	0.5	>16	8	1	8	>32	>32	2
7	C03–004	2016	TR_{34}/L98H	0.5	>16	4	1	8	32	>32	2
8	S07–008	2016	TR_{34}/L98H	0.5	>16	4	1	8	32	>32	2
9	S05–205	2017	TR_{34}/L98H	0.5	>16	4	1	8	32	>32	2
10	S05–322	2018	TR_{46}/Y121F/T289A	0.5	>16	>16	1	>16	>32	>32	>32
11	YL1	2014 (d0)	Polymorphisms¶	0.5	0.25	0.5	0.25	1	4	4	0.25
11	YL3	2014 (d100)	Polymorphisms¶	0.5	>16	8	1	8	32	>32	2
11	YL4	2015 (d134)	Polymorphisms¶	0.5	>16	8	1	8	32	>32	2
11	YL5	2015 (d165)	Polymorphisms¶	0.5	>16	2–4	0.5	2	16	16	1
11	YL6	2015 (d185)	Polymorphisms¶	0.5	>16	8	1	8	32	>32	2
12	g054m	2016 (d0)	Wild-type	0.12	>16	8	1	8	32	>32	2
12	g054L	2016 (d0)	Wild-type	0.5	0.5	0.5	0.06	0.5	1	4	0.25
12	g057m	2016 (d2)	Wild-type	0.12	>16	4	1	8	32	>32	2
12	g057L	2016 (d2)	Wild-type	0.5	0.5	0.5	0.06	0.5	1	4	0.25
Azole-susceptible isolates, n = 200#				0.5/1 (0.12–1)	0.5/0.5 (0.03–1)	0.5/1 (0.12–1)	0.12/0.25 (0.015–0.25)	ND	2/4 (1–8)	4/8 (2–8)	ND

*AMB, amphotericin B; ATCC, American Type Culture Collection; DFC, difenoconazole; ISA, isavuconazole; ITR, itraconazole; ND, not done; POS, posaconazole; PRC, prochloraz; TBC, tebuconazole; VRC, voriconazole.
†d0 and dn indicate day of and n days after the first isolation of *A. fumigatus*, respectively.
‡*Candida parapsilosis* ATCC 22019 and *A. fumigatus* ATCC MYA 3626 were used as quality control and reference strains.
§Because of good essential agreement in the azole MIC values between the CLSI and European Committee on Antimicrobial Susceptibility Testing (EUCAST) methods (*2*) and the lack of established CLSI clinical breakpoints for *A. fumigatus*, the MIC interpretive criteria for resistance in this study followed the EUCAST clinical breakpoints (i.e., >2, >2, >0.25, and >1 µg/mL) for itraconazole, voriconazole, posaconazole, and isavuconazole, respectively (*3*).The drugs for susceptibility testing were obtained from Sigma-Aldrich (https://www.sigmaaldrich.com) (AMB, ITR, VRC, POS, DFC, and PRC), Toronto Research Chemicals (https://www.trc-canada.com) (ISA), and Chem Service (https://www.chemservice.com) (TBC).
¶These isolates have F46Y/G89G/M172V/N248T/D255E/L358L/E427K/C454C polymorphisms.
#The MICs of 200 azole-susceptible isolates were used. Only 62 isolates were tested for MICs for DFC and TBC.

Appendix Table 3. Clinical characteristics of 12 patients harboring azole-resistant *Aspergillus fumigatus* isolates, Taiwan*

Patient no.	Strain no.	Age, y/ sex	Concurrent condition	Sample	*Aspergillus* disease†	Previous azole exposure	Sequential antifungal treatment (d)	Outcome	IA-related death
1‡	B44, B51	66/F	DM, HCV/cirrhosis, adrenal insufficiency	Sputum	IPA, unclassified	No	VRC (1), AMB (3)	Died	Yes
2	D007	49/M	AML, peritonitis	Nasal swab	Colonization	POS/VRC (2 wk)	MCF (33)	Died	No
3	E071, E074; E073	64/F	SLE, ESRD, bacterial septicemia, meningoencephalitis	Pleural effusion; sputum	Proven IPA with empyema	No	VRC (1), LAMB (3)	Died	Yes
4	S05–319	88/F	DM, influenza B	Sputum	Colonization	No	No	Alive	No
5‡	A31	59/M	Lung cancer, COPD, bronchiectasis	BAL	Proven IPA	No	AMB (3), POS (10), VRC (11)	Died	Yes
6	S05–122	90/F	COPD, steroid use, bronchiectasis	Sputum	Probable IPA	No	No	Died	Yes
7	C03–004	–	–	Sputum	–	–	–	–	–
8	S07–008	–	–	Ear	–	–	–	–	–
9	S05–205	76/F	COPD, steroid use, bronchiectasis, DM	Sputum	Colonization	No	No	Alive	No
10	S05–322	74/M	B cell lymphoma, COPD, HCV, CAD	Sputum	Colonization	No	No	Alive	No
11	YL1;§ YL3, YL4, YL5, YL6	36/M	HIV/AIDS	Urine; PCN	Proven IA, (renal abscess)	VRC (3 mo)	VRC (93), VRC/CAS (43), LAMB (44), LAMB/5FC (25), LAMB/AND (24)	Died	Yes
12	g054m, g054L,§ g057m, g057L§	39/M	MDS with RAEB-T, status post-HSCT with GVHD, bacterial septicemia	Sputum; BAL	Probable IPA	VRC (4 mo)	AMB (18), POS (21), AMB (13), AMB/AND (8), AND (5), AMB/AND (1), AND (4), AMB/AND (2)	Died	Yes

*AMB, conventional amphotericin B; AML, acute myeloid leukemia; AND, anidulafungin; BAL, bronchoalveolar lavage; CAD, coronary artery disease; CAS, caspofungin; COPD, chronic obstructive pulmonary disease; DM, diabetes mellitus; ESRD, end-stage renal disease; 5FC, flucytosine; GVHD, graft versus host disease; HCV, hepatitis C virus infection; HSCT, allogeneic hematopoietic stem cell transplantation; IA, invasive aspergillosis; IPA, invasive pulmonary aspergillosis; LAmB, liposomal amphotericin B; MCF, micafungin; MDS with RAEB-T, myelodysplastic syndrome with refractory anemia and excess blast in transformation; PCN, percutaneous nephrostomy; POS, posaconazole; SLE, systemic lupus erythematosus; VRC, voriconazole; –, data not available.
†Clinical data for patients harboring azole-resistant *A. fumigatus* were reviewed, and IA was classified according to the EORTC/MSG definition (*4*).
‡Three isolates (A31, B44, and B51) from 2 patients have been described in our previous report (*5*).
§YL1, g054L, and g057L were azole susceptible.

Appendix Table 4. References for 38 oversea *Aspergillus fumigatus* strains included in microsatellite-based phylogenetic analysis during a multicenter study of azole-resistant *Aspergillus fumigatus* clinical isolates, Taiwan

Strain	Reference
C485	(*6*)
C96	(*6*)
E2619	(*6*)
20643.023	(7)
20684.007	(7)
E1001	(*6*)
20677.079	(*7*)
2087 m1341.17–06–2012	(*8*)
2091 m1428.01–07–2012	(*8*)
C94	(*6*)
094411/7/50	(*8*)
Case 2–90d	(*9*)
Japan Dec 2013	(*10*)
The Netherlands 7	(*10,11*)
20643.017	(7)
2005–456307L	(6)
OKH50	(*12*)
04–202165	(*13*)
F2126	(*6*)
1042/09	(*14*)
14–148–2460	(*6*)
2107m1974.23–09–2012	(*8*)
A12519	(*8*)
R2–07–1_R	(*6*)
E454	(*15*)
Case 1–7d	(*9*)
Myc-2008–002 nr.42	(*14*)
Case 3–6d	(*9*)
CF/NL2992	(*8*)
Tanzania	(*11*)
CF/NL0682	(*8*)
VPCI651/Ei/12/2/a/3	(*8*)
CF/NL0645	(*11*)
The Netherlands 2	(*10,11*)
The Netherlands 3	(*10,11*)
C195	(*7*)
12–90032258	(*13*)
Case 4–36d	(*9*)

Appendix Table 5. Gene substitutions in azole-resistant *Aspergillus fumigatus* isolates and control strains, Taiwan*

Strain no.	Azole susceptibility (mechanism)	Gene substitutions†			
		hapE	*srbA*	*hmg1*	*erg6*
ATCC MYA-3626	S	–	V37D	–	–
ATCC 16903	S	–	V37D, S82P	H564Y	–
F2509	S	–	V37D	–	–
F02411	S	–	A865V	P212S, H564Y	–
YL1	S	–	E957D	P212S, H564Y	–
YL3	R	–	E957D	P212S, S269P, H564Y	F186V
YL4	R	–	E957D	P212S, S269P, H564Y	F186V
YL5	R	–	E957D	P212S, S269P, H564Y	–
YL6	R	–	E957D	P212S, S269P, H564Y	F186V
g054m	R	–	A865V	F262_del, H564Y	–
g057m	R	–	A865V	F262_del, H564Y	–
g054L	S	–	V37D	–	–
g057L	S	–	V37D	–	–
B44	R (TR_{34}/L98H/S297T/F495I)	–	V37D	H564Y	–
A31	R (TR_{34}/L98H)	–	V37D	–	–

*ATCC, American Type Culture Collection; del, deletion; R, resistant; S, susceptible; –, wild type.
†The *hapE*, *hmg1*, *erg6* and *srbA* genes were sequenced as described (*16–18*). The *hapE* and *srbA* sequences were compared with those of *A. fumigatus* f293; the *hmg1* and *erg6* sequences were compared with those of *A. fumigatus* A1163.

Appendix Table 6. Proposed integrated algorithm for management and control of azole-resistant aspergillosis, Taiwan*

Category	Response
Academic society	Incorporate the issue of azole resistance into national guidelines for management of aspergillosis; hold educational programs to improve diagnosis and treatment for azole-resistant aspergillosis.
Laboratory personnel	Identify clinically relevant *Aspergillus* isolates at the species complex level and confirm *A. fumigatus* by thermotolerance test (growth at 50°C) (*19*); screen for azole resistance with azole agar plates for clinically relevant *A. fumigatus* isolates and screen multiple colonies (<u><</u>5 colonies) from a single specimen (*19*); for colonies grown on any azole agar plate, perform azole MIC testing by using reference CLSI or EUCAST methods or an alternative Sensititer YeastOne assay (*19,20*); If MIC testing is not available, refer isolates to a mycology reference laboratory (*19*); prompt notification of the clinical team if azole-resistant *A. fumigatus* is suspected and identified.
Physicians	Be familiar with patient risk factors for invasive aspergillosis; obtain clinical specimens for fungal culture as possible; select empirical antifungal agents according to the updated local prevalence rate of azole resistance (*21*); antifungal susceptibility testing is recommended for *A. fumigatus* isolates from invasive diseases and should be repeated on later isolates if infection persists despite treatment (*21*); be aware of the possibility of azole-resistance in patients unresponsive to azole treatment; consider amphotericin B–based or azole/echinocandin combination therapy for azole-resistant aspergillosis (*19,21*).
Hospital environment	Segregate patients from construction or renovation, potted plants, and flowers in wards and patients' room (*22*); control the airborne dissemination of fungal spores, (e.g., barriers, containment, air handling, HEPA filters, sealed windows, sealing the area of construction or renovation activities if possible) (*23*).
Reference mycology laboratory	Identify *Aspergillus* isolates to the species level by molecular methods; confirm antifungal susceptibility of *Aspergillus* isolates with reference CLSI or EUCAST methods; perform periodic reference MIC testing of isolates of *A. fumigatus* complex (<u>></u>100 isolates) (*19*); sequence *cyp51A* genes in resistant isolates to determine the nature and trends in *cyp51A* mutation distribution (*19*); establish molecular typing methods; collect strains.
Scientists and plant pathologists	Identify the key azole fungicides that select azole-resistant *Aspergillus*; propose better fungicide application strategies to minimize resistance development (*24*).
Agricultural authority	Include azole fungicides that select azole-resistant isolates into national pesticide risk reduction programs; advise farmers to reduce culprit azole fungicide use by in rotation with alternative fungicides with different modes of action.
Governance	Update and evaluate the global situation; accredit national mycology reference laboratories; implement antifungal stewardship programs in agriculture in addition to hospitals and animal husbandry to achieve the One Health goal (*24*).

*CLSI, Clinical and Laboratory Standards Institute; EUCAST, European Committee on Antimicrobial Susceptibility Testing.

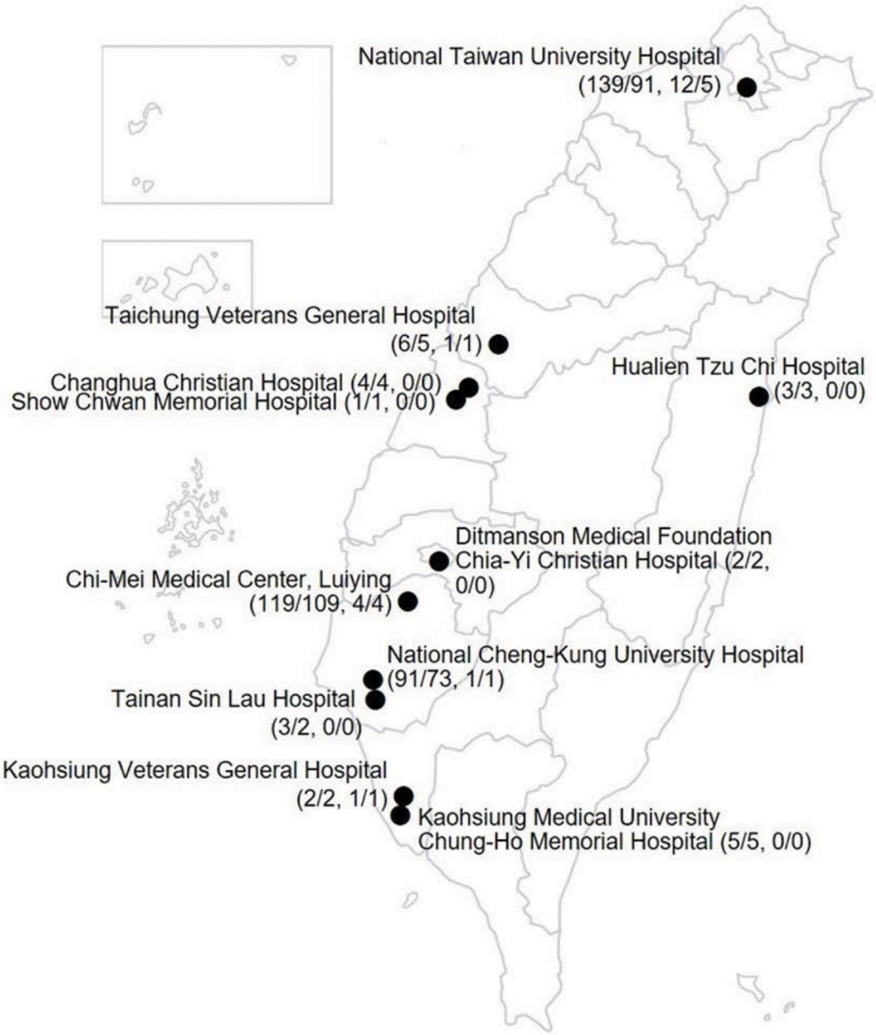

Appendix Figure 1. Distribution of participating hospitals and isolate collection areas for *Aspergillus fumigatus*, Taiwan. Values in parentheses indicate no. *A. fumigatus* isolates/no. patients and no. azole-resistant *A. fumigatus* isolates/no. patients.

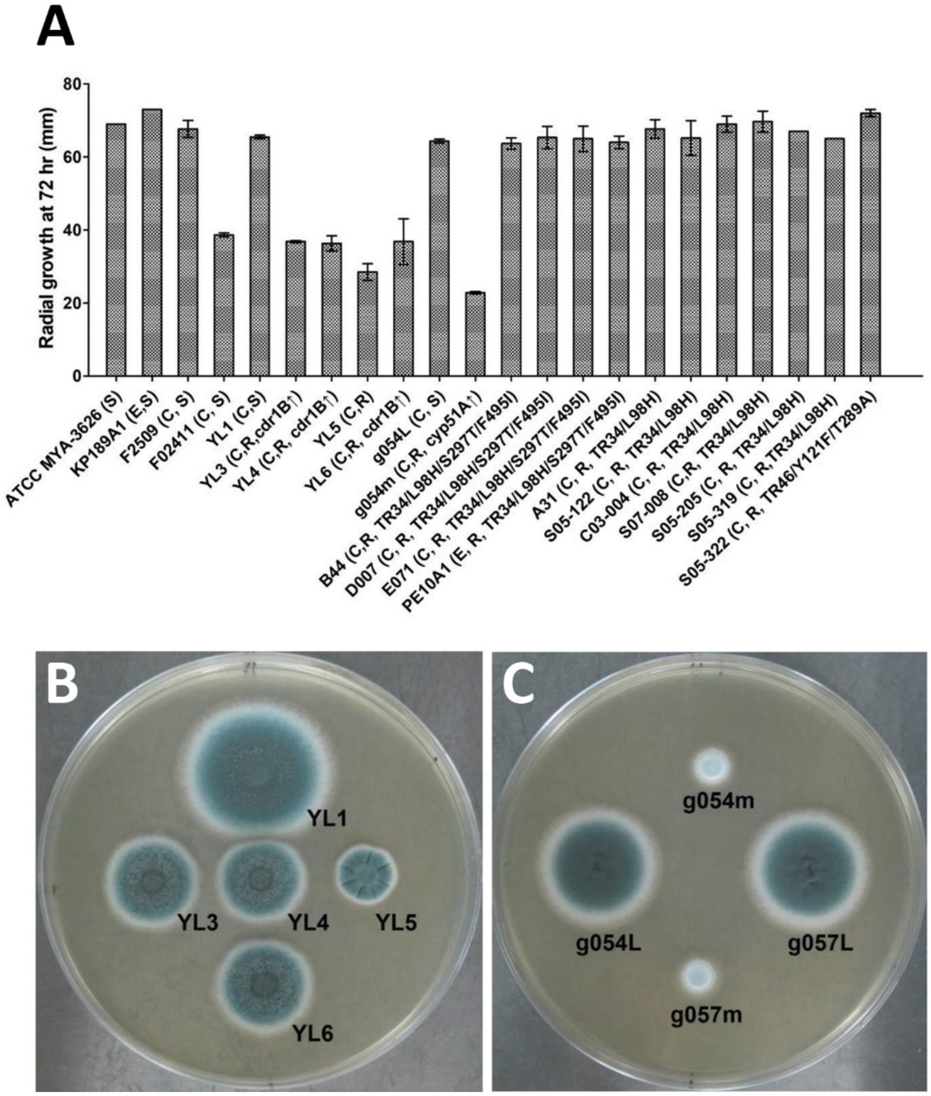

Appendix Figure 2. A) Radial growth of *Aspergillus fumigatus* isolates on Sabouraud dextrose agar plates at 35°C, Taiwan. The radius of the growing colony was measured after 72 hours of incubation. Values are the mean diameter of triplicate samples. Error bars indicate SD. Colonies of B) YL1, YL3, YL4, YL5, and YL6 from patient 11 and C) g054m, g054L, g057m, and g057L from patient 12 observed at 72 hours. C, clinical isolate; E, environmental isolate; R, azole-resistant; S, azole-susceptible; ↑, overexpression.

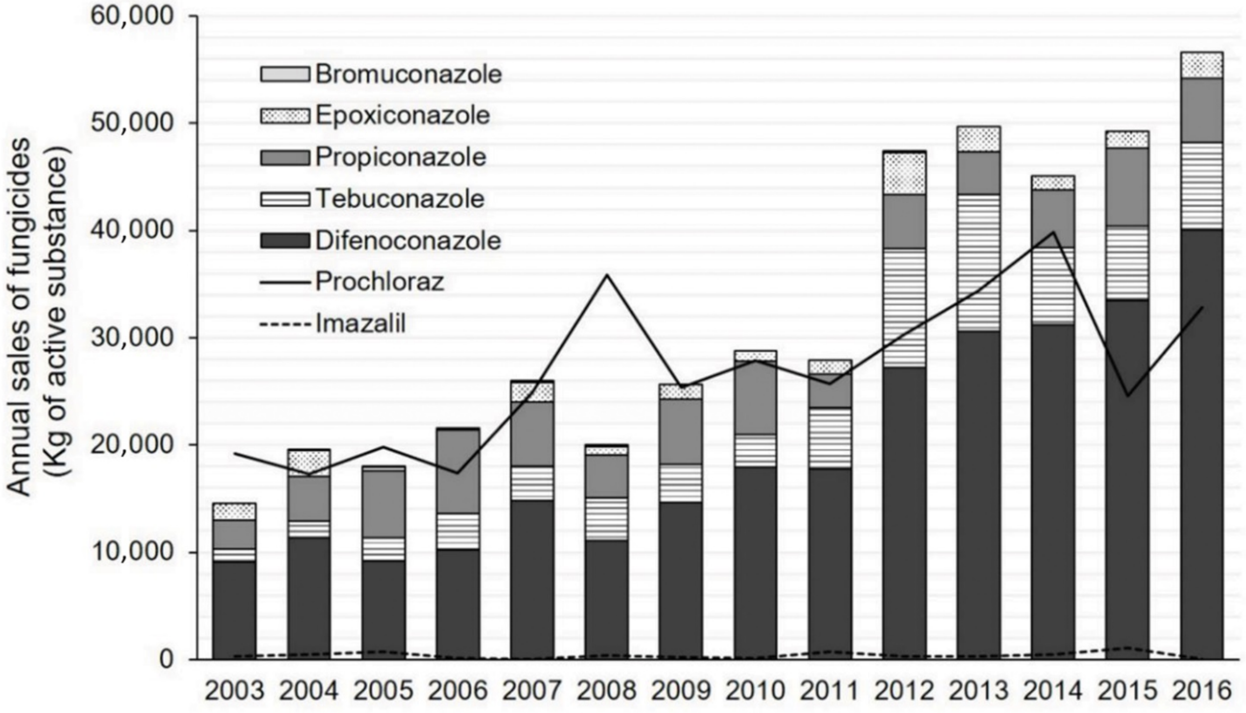

Appendix Figure 3. Annual sales of azole fungicides in Taiwan, 2003–2016. Annual sales of imidazole fungicides (imazalil and prochloraz) and triazole fungicides (bromuconazole, difenoconazole, epoxiconazole, propiconazole, and tebuconazole) are shown according to data derived from Domestic Manufacturers Production and Sale of Pesticides published by the Taiwan Crop Protection Industry Association (*25*).

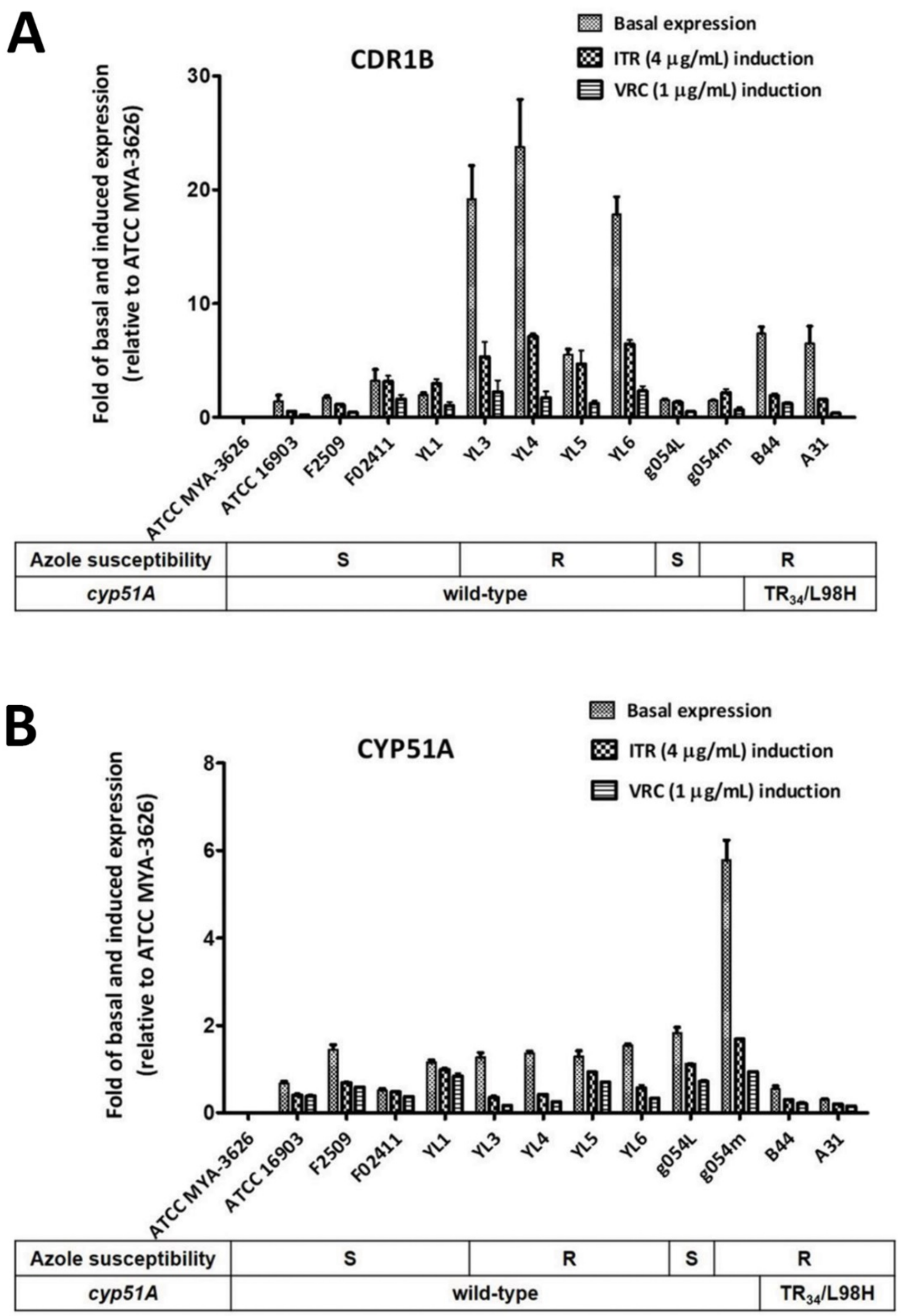

Appendix Figure 4. mRNA expression levels of A) a drug efflux transporter gene, *cdr1B*, and B) *cyp51A* in *Aspergillus fumigatus* isolates, Taiwan. Expression levels were normalized to β-tubulin levels and compared with those in *A. fumigatus* ATCC MYA-3626. Error bars indicate SD. Results for the *cyp51B* gene and other transporter genes (*AfuMDR1*, *AfuMDR2*, *AfuMDR3*, *AfuMDR4*, *atrF*, and *MFS56*) were inconclusive and are not shown. ATCC, American Type Culture Collection; ITR, itraconazole; VRC, voriconazole.

References

1. Balajee SA, Houbraken J, Verweij PE, Hong SB, Yaghuchi T, Varga J, et al. *Aspergillus* species identification in the clinical setting. Stud Mycol. 2007;59:39–46. PubMed https://doi.org/10.3114/sim.2007.59.05

2. Pfaller M, Boyken L, Hollis R, Kroeger J, Messer S, Tendolkar S, et al. Comparison of the broth microdilution methods of the European Committee on Antimicrobial Susceptibility Testing and the Clinical and Laboratory Standards Institute for testing itraconazole, posaconazole, and voriconazole against *Aspergillus* isolates. J Clin Microbiol. 2011;49:1110–2. PubMed https://doi.org/10.1128/JCM.02432-10

3. EUCAST. Subcommitee on Antimicrobial Susceptibility Testing (EUCAST-AFST). Clinical breakpoints for fungi, version 9 [cited 2020 Jan 25]. http://www.eucast.org/astoffungi/clinicalbreakpointsforantifungals

4. De Pauw B, Walsh TJ, Donnelly JP, Stevens DA, Edwards JE, Calandra T, et al.; European Organization for Research and Treatment of Cancer/Invasive Fungal Infections Cooperative Group; National Institute of Allergy and Infectious Diseases Mycoses Study Group (EORTC/MSG) Consensus Group. Revised definitions of invasive fungal disease from the European Organization for Research and Treatment of Cancer/Invasive Fungal Infections Cooperative Group and the National Institute of Allergy and Infectious Diseases Mycoses Study Group (EORTC/MSG) Consensus Group. Clin Infect Dis. 2008;46:1813–21. PubMed https://doi.org/10.1086/588660

5. Wu CJ, Wang HC, Lee JC, Lo HJ, Dai CT, Chou PH, et al. Azole-resistant *Aspergillus fumigatus* isolates carrying TR_{34}/L98H mutations in Taiwan. Mycoses. 2015;58:544–9. PubMed https://doi.org/10.1111/myc.12354

6. Chen Y, Lu Z, Zhao J, Zou Z, Gong Y, Qu F, et al. Epidemiology and molecular characterizations of azole resistance in clinical and environmental *Aspergillus fumigatus* isolates from China. Antimicrob Agents Chemother. 2016;60:5878–84. PubMed https://doi.org/10.1128/AAC.01005-16

7. Deng S, Zhang L, Ji Y, Verweij PE, Tsui KM, Hagen F, et al. Triazole phenotypes and genotypic characterization of clinical *Aspergillus fumigatus* isolates in China. Emerg Microbes Infect. 2017;6:e109. PubMed https://doi.org/10.1038/emi.2017.97

8. Steinmann J, Hamprecht A, Vehreschild MJ, Cornely OA, Buchheidt D, Spiess B, et al. Emergence of azole-resistant invasive aspergillosis in HSCT recipients in Germany. J Antimicrob Chemother. 2015;70:1522–6. PubMed https://doi.org/10.1093/jac/dku566

9. Astvad KM, Jensen RH, Hassan TM, Mathiasen EG, Thomsen GM, Pedersen UG, et al. First detection of TR_{46}/Y121F/T289A and TR_{34}/L98H alterations in *Aspergillus fumigatus* isolates from azole-naive patients in Denmark despite negative findings in the environment. Antimicrob Agents Chemother. 2014;58:5096–101. PubMed https://doi.org/10.1128/AAC.02855-14

10. Hagiwara D, Takahashi H, Fujimoto M, Sugahara M, Misawa Y, Gonoi T, et al. Multi-azole resistant *Aspergillus fumigatus* harboring Cyp51A TR_{46}/Y121F/T289A isolated in Japan. J Infect Chemother. 2016;22:577–9. PubMed https://doi.org/10.1016/j.jiac.2016.01.015

11. Chowdhary A, Sharma C, van den Boom M, Yntema JB, Hagen F, Verweij PE, et al. Multi-azole-resistant *Aspergillus fumigatus* in the environment in Tanzania. J Antimicrob Chemother. 2014;69:2979–83. PubMed https://doi.org/10.1093/jac/dku259

12. Toyotome T, Hagiwara D, Kida H, Ogi T, Watanabe A, Wada T, et al. First clinical isolation report of azole-resistant *Aspergillus fumigatus* with TR_{34}/L98H-type mutation in Japan. J Infect Chemother. 2017;23:579–81. PubMed https://doi.org/10.1016/j.jiac.2016.12.004

13. Kidd SE, Goeman E, Meis JF, Slavin MA, Verweij PE. Multi-triazole-resistant *Aspergillus fumigatus* infections in Australia. Mycoses. 2015;58:350–5. PubMed https://doi.org/10.1111/myc.12324

14. Chowdhary A, Kathuria S, Xu J, Sharma C, Sundar G, Singh PK, et al. Clonal expansion and emergence of environmental multiple-triazole-resistant *Aspergillus fumigatus* strains carrying the TR_{34}/L98H mutations in the cyp51A gene in India. PLoS One. 2012;7:e52871. PubMed https://doi.org/10.1371/journal.pone.0052871

15. Ahmad S, Khan Z, Hagen F, Meis JF. Occurrence of triazole-resistant *Aspergillus fumigatus* with TR_{34}/L98H mutations in outdoor and hospital environment in Kuwait. Environ Res. 2014;133:20–6. PubMed https://doi.org/10.1016/j.envres.2014.05.009

16. Camps SM, Dutilh BE, Arendrup MC, Rijs AJ, Snelders E, Huynen MA, et al. Discovery of a *hapE* mutation that causes azole resistance in *Aspergillus fumigatus* through whole genome sequencing and sexual crossing. PLoS One. 2012;7:e50034. PubMed https://doi.org/10.1371/journal.pone.0050034

17. Hagiwara D, Arai T, Takahashi H, Kusuya Y, Watanabe A, Kamei K. Non-*cyp51A* azole-resistant *Aspergillus fumigatus* isolates with mutation in HMG-CoA reductase. Emerg Infect Dis. 2018;24:1889–97. PubMed https://doi.org/10.3201/eid2410.180730

18. Wang HC, Huang JC, Lin YH, Chen YH, Hsieh MI, Choi PC, et al. Prevalence, mechanisms and genetic relatedness of the human pathogenic fungus *Aspergillus fumigatus* exhibiting resistance to medical azoles in the environment of Taiwan. Environ Microbiol. 2018;20:270–80. PubMed https://doi.org/10.1111/1462-2920.13988

19. Ullmann AJ, Aguado JM, Arikan-Akdagli S, Denning DW, Groll AH, Lagrou K, et al. Diagnosis and management of *Aspergillus* diseases: executive summary of the 2017 ESCMID-ECMM-ERS guideline. Clin Microbiol Infect. 2018;24(Suppl 1):e1–38. PubMed https://doi.org/10.1016/j.cmi.2018.01.002

20. Wang HC, Hsieh MI, Choi PC, Wu CJ. Comparison of the Sensititre YeastOne and CLSI M38–A2 microdilution methods in determining the activity of amphotericin B, itraconazole, voriconazole, and posaconazole against *Aspergillus* species. J Clin Microbiol. 2018;56:e00780–18. PubMed https://doi.org/10.1128/JCM.00780-18

21. Verweij PE, Ananda-Rajah M, Andes D, Arendrup MC, Brüggemann RJ, Chowdhary A, et al. International expert opinion on the management of infection caused by azole-resistant *Aspergillus fumigatus*. Drug Resist Updat. 2015;21-22:30–40. PubMed https://doi.org/10.1016/j.drup.2015.08.001

22. Freifeld AG, Bow EJ, Sepkowitz KA, Boeckh MJ, Ito JI, Mullen CA, et al.; Infectious Diseases Society of America. Clinical practice guideline for the use of antimicrobial agents in neutropenic patients with cancer: 2010 update by the Infectious Diseases Society of America. Clin Infect Dis. 2011;52:e56–93. PubMed https://doi.org/10.1093/cid/cir073

23. Kanamori H, Rutala WA, Sickbert-Bennett EE, Weber DJ. Review of fungal outbreaks and infection prevention in healthcare settings during construction and renovation. Clin Infect Dis. 2015;61:433–44. PubMed https://doi.org/10.1093/cid/civ297

24. Chowdhary A, Meis JF. Emergence of azole resistant *Aspergillus fumigatus* and One Health: time to implement environmental stewardship. Environ Microbiol. 2018;20:1299–301. PubMed https://doi.org/10.1111/1462-2920.14055

25. Taiwan Crop Protection Industry Association. Domestic manufacturers production and sale of pesticides [in Chinese]. Taipei: Taiwan Crop Protection Industry Association, 1962–2016.
